# Dermoscopy of small diameter basal cell carcinoma: a case-control study^☆^

**DOI:** 10.1016/j.abd.2023.02.005

**Published:** 2023-09-01

**Authors:** Francisca Kinzel-Maluje, Daniela González-Godoy, Pablo Vargas-Mora, Pablo Muñoz

**Affiliations:** a Dermatology Department, Faculty of Medicine, Universidad de Chile, Santiago, Chile; b Dermatology Department, Clínica Las Condes, Santiago, Chile; c Melanoma and Skin Cancer Unit, Instituto Nacional del Cáncer, Santiago, Chile

Dear Editor,

Basal Cell Carcinoma (BCC) is the most common cutaneous malignancy.^1^, ^2^, ^3^ It is not usually associated with mortality, but its local behavior can be aggressive and associated with significant morbidity.^2^, ^3^, ^4^

BCC diagnosis is usually established on clinical and dermoscopic characteristics. Dermoscopy has been proven to be useful in BCC diagnosis based mainly on pigmented and vascular structures, shiny white structures, and ulceration.^5^ Nowadays the challenge is to recognize earlier and smaller lesions, so the morbidity and risk of disfiguring scarring are minimized. To this date, there are just small data regarding the possible dermoscopic differences between smaller and larger BCCs, most of them have shown no relevant differences between these two groups.^6^, ^7^, ^8^ In this study, we sought to elucidate dermoscopic differences between smaller (≤ 4 mm diameter) and larger (> 4 mm diameter) BCCs.

Biopsy reports of BCC cases of the Pathology department of the Hospital Clínico Universidad de Chile from January 2016 to April 2021 were reviewed. Two groups were selected, first BCCs with a clinical diameter under or equal to 4 mm, and a matched control group of randomly selected cases of BCCs diameter above 4 mm. Afterwards dermoscopic pictures of the lesions were analyzed by 3 expert dermatologists independently, and discrepancies were later discussed and resolved.

Demographic and clinicopathological variables were described by frequency and percentage, while continuous variables by their mean and standard deviation. The relationship between categorical variables and size was evaluated using the Chi-Square test. When the expected frequency for a combination of variables was less than 5, Fisher's exact test was performed. Multivariate logistic regression was used to estimate adjusted Odds Ratios along with their 95% Confidence Interval. A p-value less than 0.05 was considered significant. Statistical analyzes were performed using R v4.1.3 (RCoreTeam, 2022, Vienna, Austria).

A total of 112 primary BCCs were collected, 56 small cases and 56 control cases. The mean size of small BCCs was 3.0 ± 0.9 mm, in comparison with the control group which was 10.5 ± 4.9 mm. Between the two groups, there were no statistically significant differences between mean age (≤ 4 mm diameter group: 65.2 ± 14.0 and > 4 mm diameter: 67.2 ± 12.7, p-value = 0.224) and sex. The most frequent tumor location was nose (38.4%), followed by malar (18.8%), periocular (9.8%), trunk (9.8%), scalp (5.4%), and other less predominant sites, no statistically significant differences between tumor size groups were found. Regarding histopathologic variants, cases were classified based on the worst prognostic cellular component and only the morpheaform subtype was found significantly more frequently in the cases group (7 cases [12.5%] of small BCC) and 16 cases [28.6%] in the control group, p-value = 0.035), the difference disappeared after the univariate logistic regression (OR = 4.00 95% IC [0.88‒18.2], p-value = 0.073).

The dermoscopic features of both groups are exposed in Table 1. At multivariate logistic regression, the only predictor against small BCCs that remained statistically significant was arborizing telangiectasia (OR = 4.02, 95% IC [1.43‒11.3], p-value = 0.008) (Figure 1, Figure 2). The distribution of dermoscopic features in each histological subtype comparing both size groups was analyzed in Table 2, statistically significant differences were found in micronodular BCC for concentric structures (6 [27.3%] ≤ 4 mm and 0 (0%) > 4 mm, p-value = 0.023) and in nodular BCC for short fine telangiectasia (13 [68.4%] ≤ 4 mm and 5 [29.4%] > 4 mm, p-value = 0.019).

**Table 1:** Dermoscopic features of small BCCs and the control group.

		≤ 4 mm (n = 56)	> 4 mm (n = 56)	p-value
**Arborizing telangiectasia**				0.009
	No	48 (85.7%)	36 (64.3%)	
	Yes	8 (14.3%)	20 (35.7%)	
**Short fine superficial telangiectasia**				0.449
	No	28 (50%)	32 (57.1%)	
	Yes	28 (50%)	24 (42.9%)	
**Blue grey dots**				0.242
	No	42 (75%)	47 (83.9%)	
	Yes	14 (25%)	9 (16.1%)	
**Blue grey globules**				0.541
	No	40 (71.4%)	37 (66.1%)	
	Yes	16 (28.6%)	19 (33.9%)	
**Blue grey ovoid nests**				0.654
	No	44 (78.6%)	42 (75%)	
	Yes	12 (21.4%)	14 (25%)	
**Spoke wheel structures**				0.751
	No	50 (89.3%)	51 (91.1%)	
	Yes	6 (10.7%)	5 (8.9%)	
**Concentric structures**				0.039
	No	46 (82.1%)	53 (94.6%)	
	Yes	10 (17.9%)	3 (5.4%)	
**Leaf-like areas**				0.257
	No	41 (73.2%)	46 (82.1%)	
	Yes	15 (26.8%)	10 (17.9%)	
**Shiny white structures**				0.315
	No	40 (71.4%)	35 (62.5%)	
	Yes	16 (28.6%)	21 (37.5%)	
**MAY globules**^a^				0.405
	No	50 (89.3%)	47 (83.9%)	
	Yes	6 (10.7%)	9 (16.1%)	
**Erosion or ulceration**				0.025
	No	44 (78.6%)	33 (58.9%)	
	Yes	12 (21.4%)	23 (41.1%)	

a Multiple aggregated yellow-white (MAY) globules.

**Figure 1:**
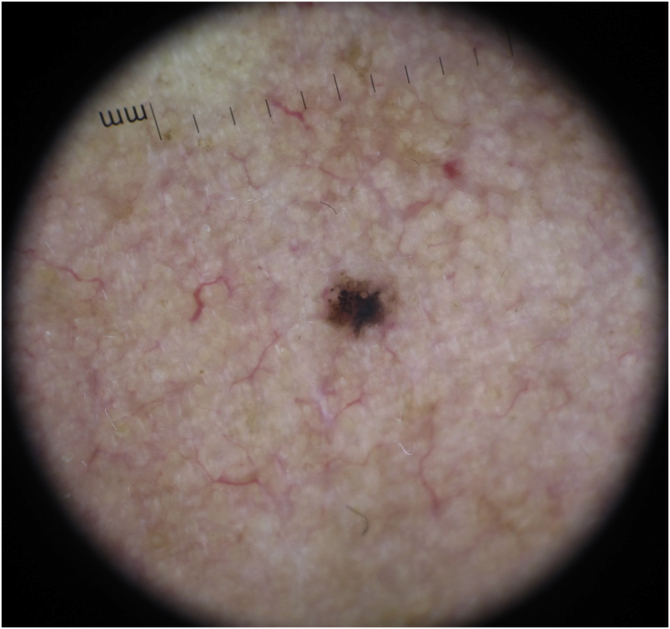
Small basal cell carcinoma, 1.5 mm in diameter, characterized by leaf-like structures and brown dots

**Figure 2:**
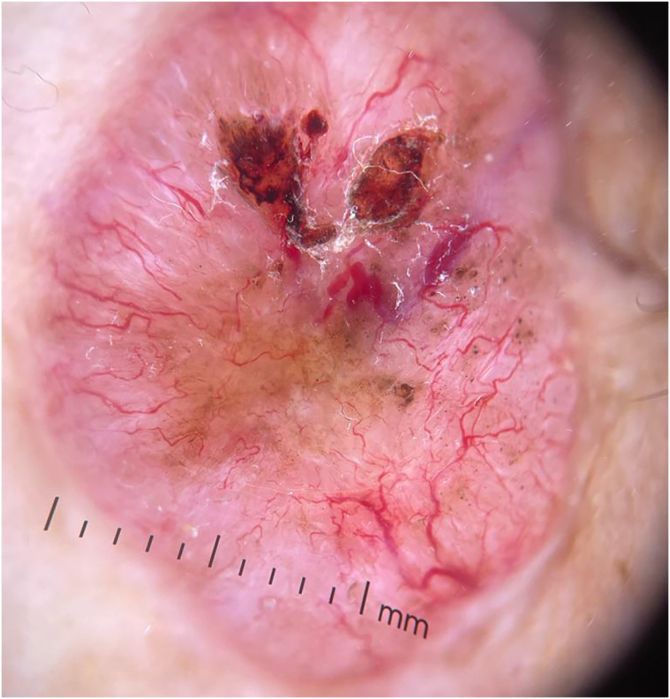
A BCC approximately 20 mm in diameter, characterized by prominent arborizing telangiectasia, ulceration, and brown structures

**Table 2:** Distribution of dermoscopic features in different histological subtypes of both tumor size groups.

Characteristics	Superficial			Micronodular			Morpheaform			Nodular		
	≤ 4 mm, (n = 7)^a^	> 4 mm, (n = 4)^a^	p-value^b^	≤ 4 mm, (n = 22)^a^	> 4 mm, (n = 19)^a^	p-value^c^	≤ 4 mm, (n = 7)^a^	> 4 mm, (n = 16)^a^	p-value^b^	≤ 4 mm, (n = 19)^a^	> 4 mm, (n = 17)^a^	p-value^c^
Arborizing telangiectasia	0 (0.0%)	0 (0.0%)		6 (27.3%)	7 (36.8%)	0.5	0 (0.0%)	7 (43.8%)	0.057	2 (10.5%)	6 (35.3%)	0.11
Short fine superficial telangiectasia	1 (14.3%)	2 (50.0%)	0.5	7 (31.8%)	9 (47.4%)	0.3	6 (85.7%)	8 (50.0%)	0.2	13 (68.4%)	5 (29.4%)	0.019
Blue grey dots	2 (28.6%)	0 (0.0%)	0.5	5 (22.7%)	6 (31.6%)	0.5	1 (14.3%)	0 (0.0%)	0.3	6 (31.6%)	3 (17.6%)	0.5
Blue grey globules	1 (14.3%)	0 (0.0%)	>0.9	9 (40.9%)	10 (52.6%)	0.5	0 (0.0%)	4 (25.0%)	0.3	6 (31.6%)	5 (29.4%)	0.9
Blue grey ovoid nests	2 (28.6%)	0 (0.0%)	0,5	4 (18.2%)	7 (36.8%)	0.2	0 (0.0%)	2 (12.5%)	>0.9	6 (31.6%)	5 (29.4%)	0.9
Spoke wheel structures	4 (57.1%)	3 (75.0%)	>0.9	2 (9.1%)	2 (10.5%)	>0.9	0 (0.0%)	0 (0.0%)		0 (0.0%)	0 (0.0%)	
Concentric structures	2 (28.6%)	1 (25.0%)	>0.9	6 (27.3%)	0 (0.0%)	0,023	1 (14.3%)	1 (6.2%)	0,5	1 (5.3%)	1 (5.9%)	>0.9
Leaf-like areas	4 (57.1%)	3 (75.0%)	>0.9	7 (31.8%)	3 (15.8%)	0,3	1 (14.3%)	3 (18.8%)	>0.9	3 (15.8%)	1 (5.9%)	0.6
Shiny white structures	0 (0.0%)	0 (0.0%)		9 (40.9%)	8 (42.1%)	>0.9	2 (28.6%)	6 (37.5%)	>0.9	4 (21.1%)	7 (41.2%)	0.2
MAY globules^d^	0 (0.0%)	0 (0.0%)		2 (9.1%)	1 (5.3%)	>0.9	0 (0.0%)	3 (18.8%)	0,5	3 (15.8%)	5 (29.4%)	0.4
Erosion or ulceration	1 (14.3%)	2 (50.0%)	0.5	4 (18.2%)	8 (42.1%)	0.093	3 (42.9%)	7 (43.8%)	>0.9	4 (21.1%)	6 (35.3%)	0.5

a n (%).

b Fisher's exact test.

c Pearson's Chi-Squared test; Fisher's exact test.

d Multiple aggregated yellow-white (MAY) globules.

Some other data about the possible dermoscopic differences between smaller and larger BCCs have been already published. Longo et al.^6^ described 87 cases of BCC under 5 mm in diameter and matching controls above 5 mm, they did not find significant differences in dermoscopic criteria, except ulceration and multiple small erosions, that were more frequently found in large BCCs. Sanchez-Martin et al.^7^ published a series of 100 BCC with a diameter under 6 mm. Of these tumors, 77% were easily diagnosed by the classic Menzies method, which did not include newer known dermoscopic findings of BCC. A subgroup of 3 mm or less diameter tumors showed similar findings. Regarding just pigmented tumors, Takahashi et al.^8^ described a small series of 11 pigmented BCC under 7 mm, showing the same classic dermoscopic findings in BCC sized in diameter between 4 and 6 mm and 3 mm or under. Our findings suggest a similar picture, but another predictive feature not previously described as significant was the presence of arborizing telangiectasia, favoring bigger tumors. We did not find literature that explains this difference, but it could be related to an increased need for blood in bigger tumors, as some authors have suggested happens in nodular BCC compared to superficial BCC.^9^ When analyzing each histological subtype, dermoscopic differences were found between smaller and bigger BCCs in micronodular tumors for concentric structures and in nodular tumors for short fine telangiectasia, to our knowledge this has not been previously described.

In summary, small BCCs share most of the known BCC dermoscopic features, for now, just a few differences have been found, but its core characteristics remain the same. Therefore, it seems that dermoscopy is a valuable tool to identify small BCCs and offer patients treatment in the early stages, reducing morbidity associated with this disease.

## Financial support

None declared.

## Authors' contributions

Francisca Kinzel-Maluje: The study concept and design; data collection, or analysis and interpretation of data; writing of the manuscript or critical review of important intellectual content; data collection, analysis, and interpretation; effective participation in the research guidance; intellectual participation in the propaedeutic and/or therapeutic conduct of the studied cases; critical review of the literature; final approval of the final version of the manuscript.

Daniela González-Godoy: Data collection, or analysis and interpretation of data; writing of the manuscript or critical review of important intellectual content; data collection, analysis and interpretation; effective participation in the research guidance; intellectual participation in the propaedeutic and/or therapeutic conduct of the studied cases; final approval of the final version of the manuscript.

Pablo Vargas-Mora: The study concept and design; data collection, or analysis and interpretation of data; writing of the manuscript or critical review of important intellectual content; data collection, analysis and interpretation; effective participation in the research guidance; intellectual participation in the propaedeutic and/or therapeutic conduct of the studied cases; critical review of the literature; final approval of the final version of the manuscript.

Pablo Muñoz: Data collection, or analysis and interpretation of data; data collection, analysis and interpretation; intellectual participation in the propaedeutic and/or therapeutic conduct of the studied cases; final approval of the final version of the manuscript.

## Conflicts of interest

None declared.
